# Supplementary figures and images for: Oregano essential oil vapour prevents Plasmopara viticola infection in grapevine (Vitis Vinifera) and primes plant immunity mechanisms
Source: PLoS One. 2019 Sep 27;14(9):e0222854. doi: 10.1371/journal.pone.0222854 (PMC6764689; doi:10.1371/journal.pone.0222854)

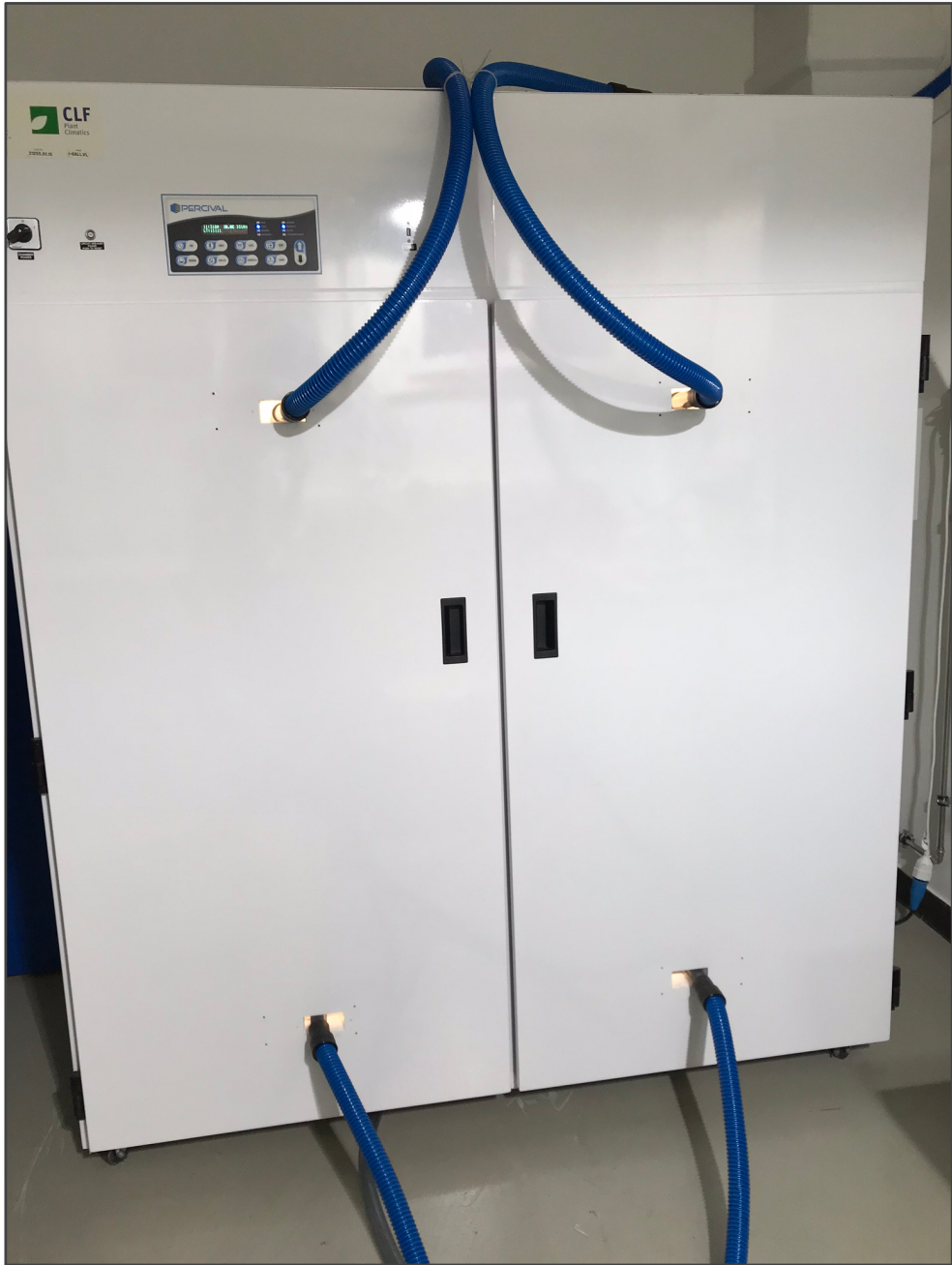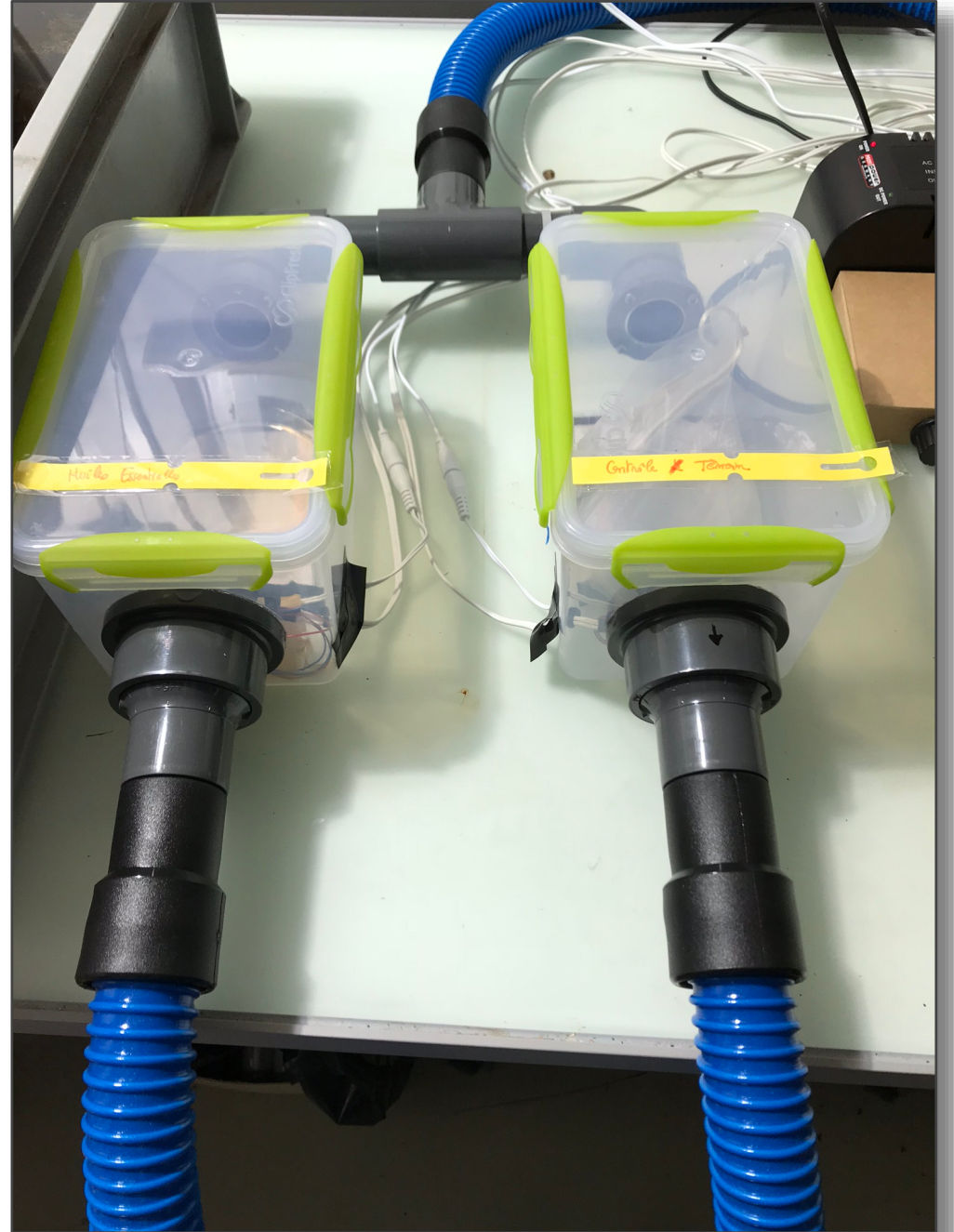

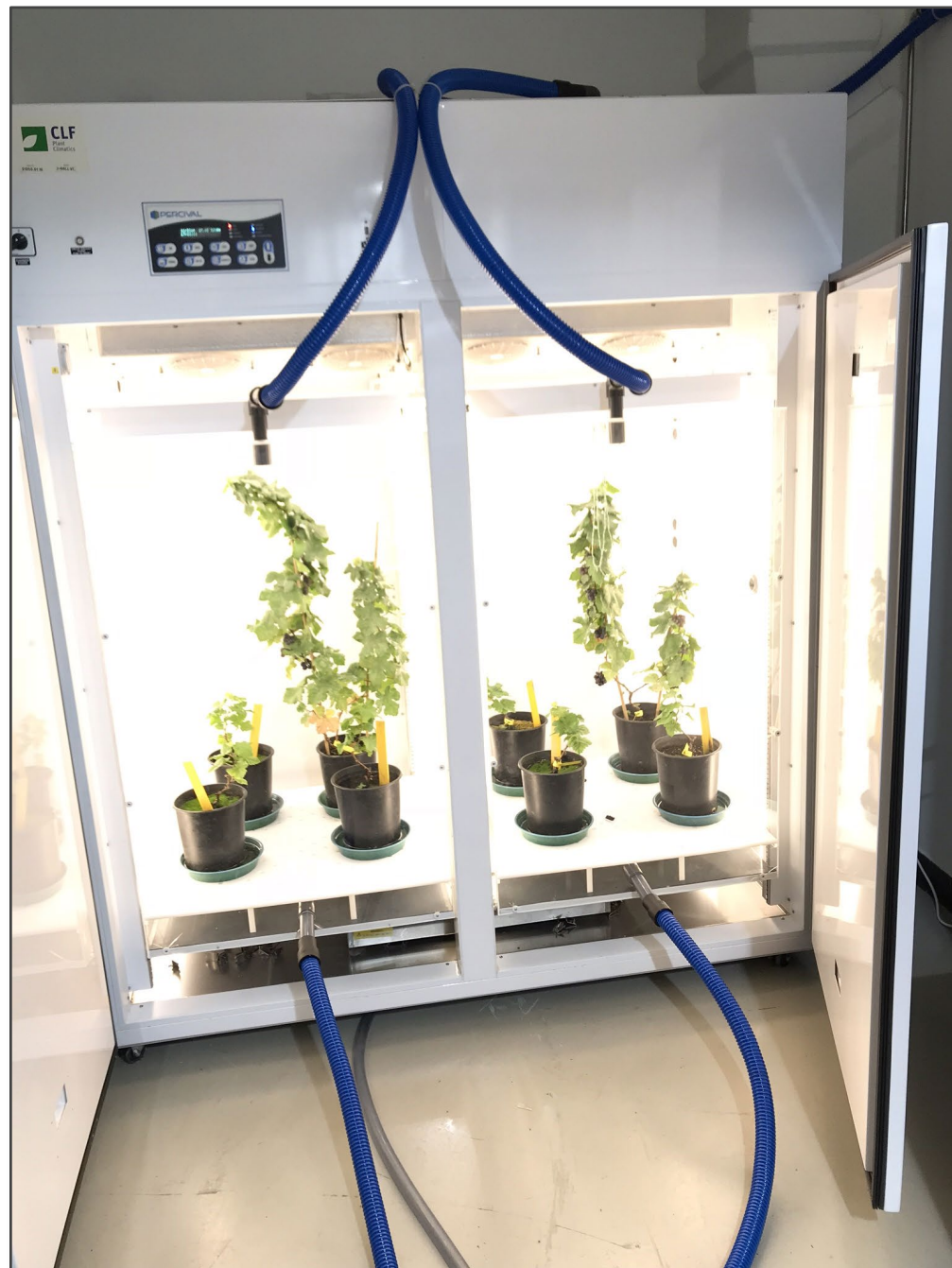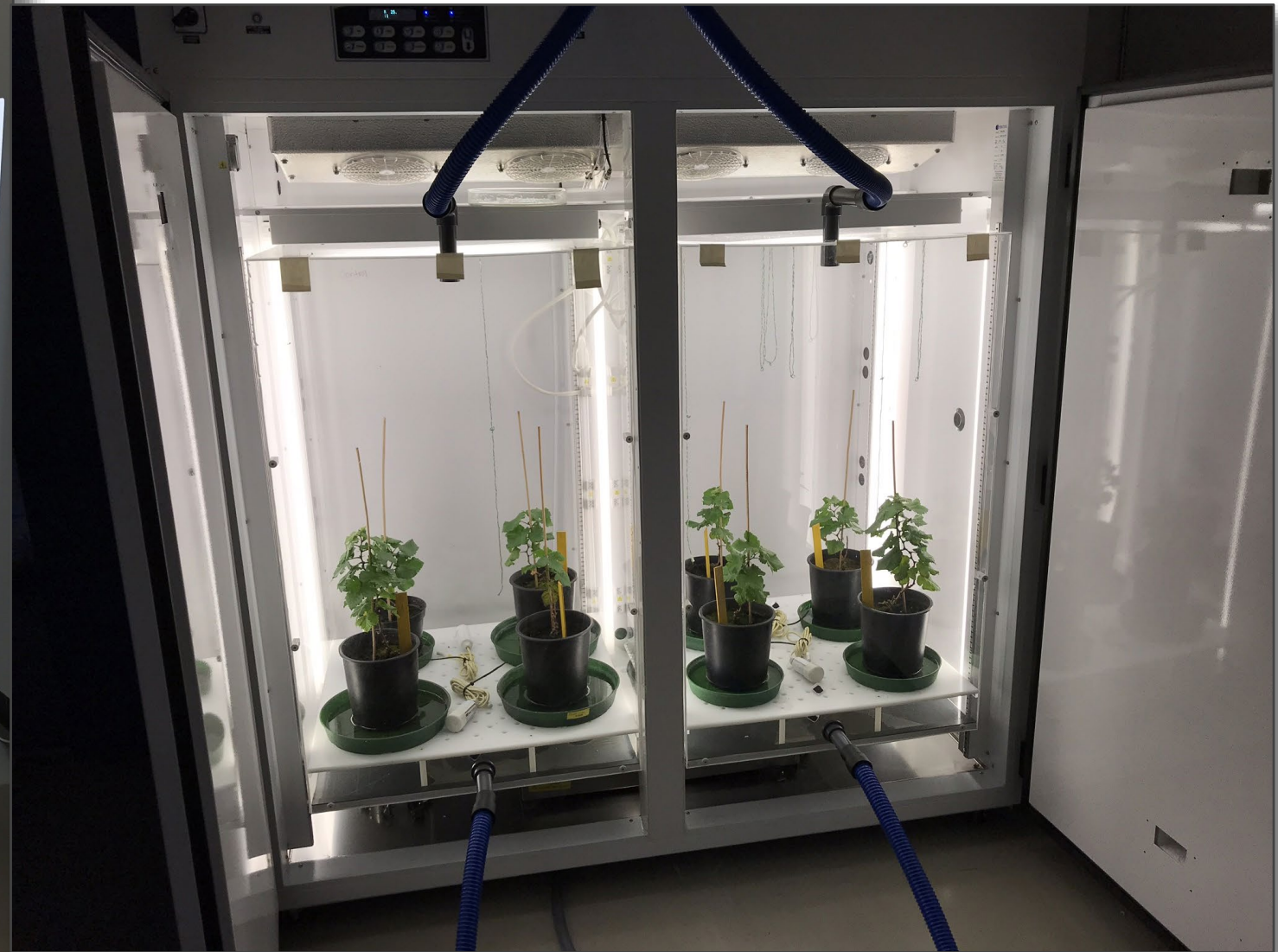

Supplement: S1 File — (PDF) [file pone.0222854.s001.pdf]

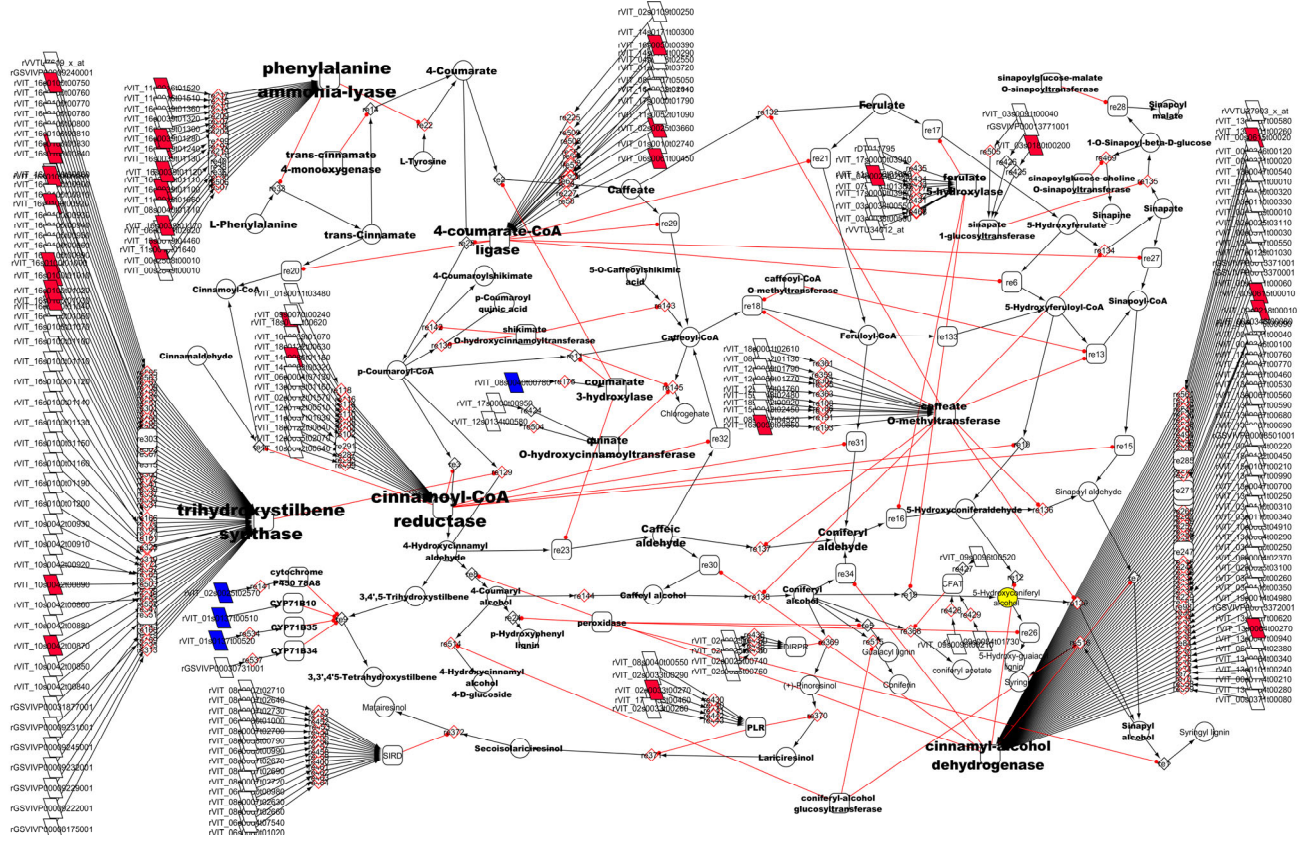

Supplement: S1 Fig — Blue: repressed, red: induced transcripts by 24h EO vapour treatment. Parallelograms: RNA; Round rectangles: proteins; Ellipse: simple molecules; Diamonds: state transitions, transcription and translation. (PDF) [file pone.0222854.s002.pdf]
